# Systemic Lupus Erythematosus: Molecular Mimicry between Anti-dsDNA CDR3 Idiotype, Microbial and Self Peptides—As Antigens for Th Cells

**DOI:** 10.3389/fimmu.2015.00382

**Published:** 2015-07-28

**Authors:** Kristin Aas-Hanssen, Keith M. Thompson, Bjarne Bogen, Ludvig A. Munthe

**Affiliations:** ^1^Department of Immunology, Centre for Immune Regulation, Institute of Clinical Medicine, University of Oslo, Oslo, Norway; ^2^KG Jebsen Centre for Influenza Vaccine Research, Institute of Clinical Medicine, University of Oslo, Oslo, Norway

**Keywords:** molecular mimicry, systemic lupus erythematosus, idiotypes, B cells, Th cells, B cell receptor, complementarity determining region 3, antigen presentation

## Abstract

Systemic lupus erythematosus (SLE) is marked by a T helper (Th) cell-dependent B cell hyperresponsiveness, with frequent germinal center reactions, and gammaglobulinemia. A feature of SLE is the finding of IgG autoantibodies specific for dsDNA. The specificity of the Th cells that drive the expansion of anti-dsDNA B cells is unresolved. However, anti-microbial, anti-histone, and anti-idiotype Th cell responses have been hypothesized to play a role. It has been entirely unclear if these seemingly disparate Th cell responses and hypotheses could be related or unified. Here, we describe that H chain CDR3 idiotypes from IgG^+^ B cells of lupus mice have sequence similarities with both microbial and self peptides. Matched sequences were more frequent within the mutated CDR3 repertoire and when sequences were derived from lupus mice with expanded anti-dsDNA B cells. Analyses of histone sequences showed that particular histone peptides were similar to VDJ junctions. Moreover, lupus mice had Th cell responses toward histone peptides similar to anti-dsDNA CDR3 sequences. The results suggest that Th cells in lupus may have multiple cross-reactive specificities linked to the IgVH CDR3 Id-peptide sequences as well as similar DNA-associated protein motifs.

## Introduction

The initiation of autoimmune responses is associated with infection and the development of a gradually evolving T cell and B cell autoreactive response toward self-proteins. Molecular mimicry is the concept that similarities of microbial peptides to self-peptides can allow expansion of microbial specific T cells that can cross react to similar self-peptides (1, 2). Molecular mimicry has been suggested to play a part in diverse autoimmune diseases such as multiple sclerosis, rheumatoid arthritis, diabetes, stromal keratitis, myocarditis, and inflammatory bowel disease (1, 2). It was originally believed that TCR binding to peptide:MHC (pMHC) was dependent upon stringent requirements for amino acid (aa) identity of the T cell contact residues. It is increasingly clear that degeneracy in both the TCR (3, 4) and MHC (5) peptide-binding motifs as well as interchangeable aa with similar properties in the TCR-exposed aa motifs (4, 6, 7) reduce this sequence-specific requirement (1).

Anti-dsDNA B cells and high titers of nephrotoxic anti-dsDNA autoantibodies are hallmarks of systemic lupus erythematosus (SLE) (8). Molecular mimicry has not been studied extensively in SLE, but a role for Th cells is well-established; it is clear that the expansion of autoreactive B cell requires pathogenic Th cells (8–11). However, the antigen specificity of the Th cells has been unclear. Candidate antigens include peptides derived from nucleic acid-associated proteins such as small nuclear riboprotein (12), histones (13), or similar proteins derived from bacteria or viruses (14). Hence, anti-DNA/RNA B cells could bind and endocytose nucleic acids as well as DNA- or RNA-associated proteins. Th cells that are specific for peptides derived from such self proteins or similar peptides derived from pathogens could thereafter collaborate with the B cells.

Another candidate for Th cell antigen is variable regions of antibody, i.e., V region idiotypes, Id. Th cells can have Id:MHC-specific TCR (15–33). It has been demonstrated that anti-dsDNA B cells can present Id:MHC class II to Id-specific Th cells, undergo the germinal center reaction, differentiate, secrete autoantibodies, and cause vasculitis and nephritis (28, 30, 31, 33). Moreover, Id-specific Th cell responses increased with disease severity in lupus mice, and disease was aggravated by injection of Id-peptide analogs (22).

We here hypothesize that these disparate suggestions for Th cell antigen in lupus are examples drawn from a network of peptide mimics. RNA/DNA-associated proteins and anti-dsDNA antibodies both have positively charged (cationic) nucleotide-binding motifs. Thus, Th cells that are specific for cationic microbial peptides may cross react with B cells presenting cationic self peptides or Id peptides from anti-DNA BCR. If so, Th cells could support B cells that present such peptide mimics thereby allowing autoantibody secretion.

We therefore aimed to analyze if the seemingly dissimilar Th cell specificities for cationic CDR3 idiotypes and cationic DNA-binding peptides could constitute networks of molecular mimics for Th cells in SLE. We chose to compare heavy chain junctions (IgVH CDR3 peptides) from mice with lupus with proteomes from microbes and mouse. The CDR3 of the heavy chain has the highest potential for novel peptides, both in terms of N region diversity (V–N1–D–N2–J) as well as the option of alternate reading frames of the D gene segment, and as a site for mutations.

We first compared IgVH CDR3 idiotypes from lupus mice with bacterial proteomes, thereafter viral proteins and self proteins. Using bioinformatics analyses, we found a surprisingly high rate of matches between CDR3 sequences and microbial proteomes as well as with self proteins, including histones. We also found that mice suffering from Id-driven lupus (with high levels of anti-dsDNA responses) developed Th cell responses toward anti-dsDNA mAbs with CDR3 sequences that resembled histones, suggesting epitope spreading involving cationic peptide mimics including idiotypes and self proteins.

## Materials and Methods

### Mice

Mice were transgenic for both the λ2^315^ Ig L-chain derived from the MOPC315 myeloma, as well as a TCR αβ transgene specific for the Id(λ2^315^)-peptide presented on I-E^d^ MHC class II molecules (30, 31). These double transgenic mice are called DTG mice herein. The Norwegian Animal Research Authority approved the experiments.

### IgVH sequence processing and analysis

The following control data sets were downloaded from NCBI^1^. 1) control VH sequences from the BALB/c, retrieved with the search term “V region immunoglobulin heavy chain BALB,” 2) Sequences derived from splenic L2–TG mice IgG^+^ B cells as deposited (34), 3) neonatal liver B cell IgVH sequences as deposited (35).

Sequences were analyzed with the IMGT/HighV-QUEST version: 1.1.2 or IMGT/HighV-QUEST version: 3.2.30 tools^2^ and compared to the IMGT/V-QUEST reference directory release: 201310-4 (14 March 2013) (36). IgVH region family identification and clonality analysis were performed on the statistics module of IMGT/HighV-QUEST. Statistics were reported only for unique sequences (36). Translated aa sequences were further analyzed in Excel (Microsoft), aa in individual positions were counted with the “countif” function.

### Search for similarities between CDR3 and microbial, self and viral proteomes

**Figure 1:**
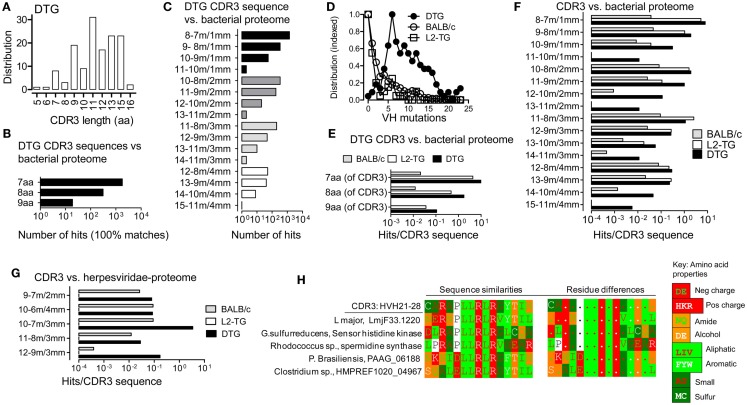
**Analysis of similarities between CDR3 sequences and the microbial proteome**. **(A)** IgVH CDR3 length distribution of sequences from DTG mice with end stage lupus. **(B,C,E–H)**, Results from Blastp analysis of 15mer CDR3 peptides vs. microbial proteomes and G, *Herpesviridae* proteome. **(B)** DTG IgG IgVH CDR3 peptides vs. microbial proteome: Numbers of hits with 100% matches with contiguous microbial sequences are shown (i.e. alignments without gaps). **(C)** DTG IgG IgVH CDR3 vs. microbial proteome: Number of hits in the indicated categories of length with matched/mismatched aa. For example: a 12 aa stretch that includes 10 matches (m) and 2 mismatches (mm) is labeled “12–10 m/2 mm.” **(D)** Mutations per IgVH sequence (not including CDR3) in the three sequence data sets: BALB/c, DTG IgG, L2-TG IgG (see Materials and Methods). The highest frequency of mutations for each data set is normalized to 1 on the *Y* axis. **(E)** Exact matches normalized to the number of input sequences in DTG vs. L2-TG IgG data sets. **(F)** Hits with matches/mismatches [as denoted in **(C)**] normalized to the number of input sequences in DTG, L2-TG, and BALB/c data sets. **(G)** Hits with matches/mismatches as in **(F)**, compared to *Herpesviridae* proteome, normalized to the number of input sequences. **(H)** Example of one DTG anti-dsDNA IgVH CDR3 sequence and similar microbial sequences. An additional hit from outside the microbial databases is also shown, from the *Leishmania major* protozoa. Sequences similarities between IgVH CDR3 and proteins are shown in the left panels. Amino acids are color coded according to charge (Negative: D, E; positive: H, K, R), or the chemical properties of side chains (i.e. amide: N, Q; alcohol: S, T; aliphatic: L, I, V; aromatic: F, Y, W; small size: A, G; sulfur atom: M, C; or other: P), see key for color code. In the right panels, differences in the sequences are marked by aa symbols when such aa do not belong to the same chemical group as the most frequent aa at the corresponding position of the comparison set.

**Figure 2:**
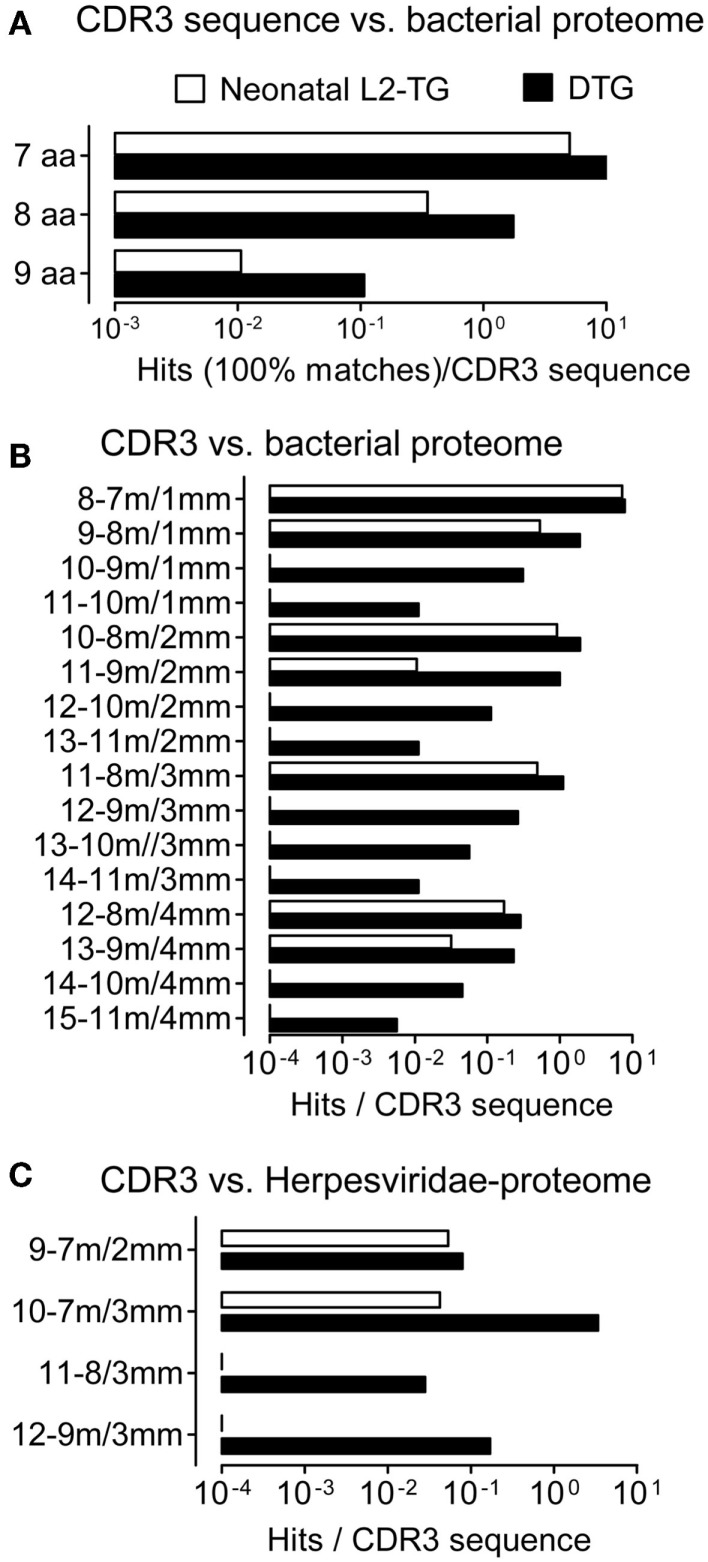
**Analysis of CDR3 from IgVH of neonatal sequences, role of somatic mutations**. We compared IgVH CDR3/microorganism matches in two data sets derived from the highly mutated DTG sequences and neonatal sequences from L2-TG mice. **(A)** Exact matches per sequence are shown in contiguous microbial sequences. **(B)** Hits matches/mismatches in the microbial proteome are shown (aa length-matches/mismatches, denoted as in Figure 1). **(C)** Matches including mismatches compared to *Herpesviridae* proteome, hits normalized to the number of input sequences are shown.

Only CDR3 sequences from 11–16 aa length were included in the analysis, sequences with CDR3 length 11, 12, 13, and 14 included FR3 aa (4, 3, 2, and 1 aa, respectively) so that 15mers (or 16mers) were used. CDR3 sequences were analyzed on the Basic Local Alignment Search Tool (BLAST) server^3^ with the Blastp suite (protein–protein BLAST) and search parameters were adjusted to search for a short input sequence. CDR3 peptides were compared to 1) the microbial genomes (BLOSUM62 matrix), constituting mostly bacterial species (see list in the Supplementary Material); 2) the mouse proteome utilizing the mouse Refseq genome; 3) CDR3 sequences were also compared to non-redundant databases (GenBank CDS translations + PDB + SwissProt + PIR + PRF excluding environmental samples from WGS projects). Resulting sequence matches were manually screened for sequences of microorganism origin; 4) sequence analyses were performed against viral databases at the Viral Bioinformatics Resource Center^4^. Figures 1 and 2 show analysis of sequences compared to the *Herpesviridae* protein database (11,887 sequences; 5,156,626 total letters, database posted June 14, 2012).

Alignments and matches were performed by registering only contiguous, i.e. non-gap’-ed sequences. aa equivalence was scored according to BLAST algorithms.

To analyze similarities between previously published VH CDR3 sequences and histones, we made artificial sequence files where variously truncated DNA-binding motifs from histones (H1, H2A, H4) were inserted into sequences starting with the aa CAR and terminating with four to six aa from FR4. Artificial sequence files were screened for matches with Ig sequences. In the Blast analyses, only contiguous (ungapped) matches were included. Sequence visualization and generation of similarity and difference plots were done with the GeneDoc program^5^.

### Th cell culture and *In vitro* assays

Th cells (5 × 10^4^/well) from lymph nodes of DTG mice, Id^+^ single transgenic mice (L2-TG) and BALB/c were mixed with 2000 Rad-irradiated BALB/c splenocytes (5 × 10^5^/well) and 16/17mer peptides derived from histones as indicated (see below), [^3^H]TdR was added on day 3, proliferation was measured as counts on a TopCount NXT Scintillation Counter (PerkinElmer) on day 6.

Th cell lines from lymph nodes of DTG mice, L2-TG mice and BALB/c were stimulated by irradiated BALB/c splenocytes and indicated peptides, were restimulated in 10 day cycles, IL-2 was first provided on first re-stimulation. Th cell lines from DTG mice were negative for the clonotype specific mAb GB113 (30, 31) that stains the transgenic Id-specific TCR and did not respond to Id (λ2^315^)-peptide.

DNA-binding histone sequences were identified, peptides were synthesized by Think Peptides. Histone H2A superfamily: called HisH2A-epitope 1 (ep1), ^28^VGRVHRLLRKGNYAERV^44^ (17mer), HisH2A-ep2: ^66^LAGNAARDNKKTRIIPR^82^ (17mer). Histone H4 (HisH4-ep1): ^35^IRRLARRGGVKRISGL^50^ (16mer). HisH4-ep2: ^70^AVTYTEHAKRKTVTAM^85^ (16mer), histone H1 family, HisH1: ^75^KNNSRIKLGLKSLVSK^90^ (16mer).

## Results

### CDR3 sequences from Anti-DNA B cells from lupus mice show multiple similarities with microbial sequences

We have previously described IgVH sequences from lupus prone mice suffering from Id-driven lupus (33). Utilizing a data set of 176 sequences with average of 11 IgVH CDR3 aa (Figure 1A), we compared CDR3 sequences with microbial sequences utilizing Blastp (microbial sequences include non-redundant data from prokaryotic genome sequencing projects, but not viruses or eukaryotic pathogens such as protozoa and fungi), see Materials and Methods. Because T cells recognize linear epitopes, only contiguous (i.e. non-gap’-ed) matches were analyzed. With this approach, we found frequent hits as 7, 8, or 9 contiguous matched aa could be found in the microbial proteomes (Figure 1B). Moreover, when analyzing sequences in terms of matches including mismatches, further hits were found (Figure 1C), for example the DTG IgVH CDR3 sequences data set had 55 hits in the category 10 contiguous aa with 9 matches (m), 1 mismatch (mm), denoted 10–9 m/1 mm in the figures.

### CDR3 sequences from B cells with a high number of VH mutations are more likely to match microbial sequences

We proceeded to compare the IgVH sequences from DTG lupus mice with sequences derived from IgG^+^ B cells from single λ2^315^ transgenic (L2-TG) mice (34). These mice are healthy and have B cells expressing the same λ2^315^ transgene as the current DTG lupus mice. As another unbiased control we used >2000 BALB/c sequences (all, deposited and annotated BALB/c sequences, see Materials and Methods). The DTG mice have a high mutation rate in the VH gene segment compared to the other two data sets, but both L2-TG and BALB/c included mutated sequences (Figure 1D).

Continuing the analysis, we blasted each data set toward the microbial proteome and normalized the rate of hits to the number of analyzed input sequences in each of the three groups. We found that IgVH CDR3 from DTG lupus mice with the highest rate of VH gene mutations had a 1.4–3.7 increased frequency of exact matches (DTG vs. L2-TG, Figure 1E). A similar tendency was seen in sequences with few mismatched aa (Figure 1F), for example the category with 10 contiguous aa consisting of 9 matches and 1 mismatch (10–9 m/1 mm), had 0.3 hits per IgVH CDR3 sequence from the DTG data set, 0.04 per IgVH CDR3 in the sequences from the L2-TG mice and 0.009 per IgVH CDR3 in the sequences from the BALB/c mice. L2-TG IgG sequences had a higher hit rate than BALB/c sequences, a finding that may relate to a higher level of marginal zone B cells in the L2-TG mice (34).

To extend the analysis to viral proteomes, we compared the IgVH CDR3 data sequence data sets with the proteome from *Herpesviridae*, a large family of DNA viruses (see Materials and Methods). With this analysis, a similar tendency was seen. For example, DTG IgVH CDR3 had on average 3.4 hits per IgVH CDR3 sequence to herpes viral proteins in the category of sequences with 7 aa matches and 3 mismatches (10–7 m/3 mm). The corresponding analysis on sequences from BALB/c mice (10–7 m/3 mm) revealed 0.09 hits per CDR3 sequence (Figure 1G). L2-TG sequences had no hits in the categories shown.

In addition to the analysis above, 10 DTG anti-dsDNA IgVH CDR3 sequences were blasted toward all proteome sequences (i.e. an unrestricted, non-redundant protein sequence Blastp analysis). Results were manually screened for examples of hits toward non-bacterial pathogens (e.g. protozoa, fungi). Examples of the matches between one particular DTG anti-dsDNA IgVH CDR3 sequence and microbial proteomes can be seen in Figure 1H. This figure also includes examples of hits in the proteomes of two eukaryotic pathogens, the *Leishmania major* protozoa (12–10 m/2 mm); and the *Paracoccidioides brasiliensis* fungus (10–10 m/0 mm) – these latter results as obtained by Blastp, and manual screening.

### CDR3 sequences from neonatal L2-TG mice have reduced matches with microbial proteomes

The analyses above were based on sequences from adult mice, including sequences with mutations (see Figure 1D). To analyze the impact of antigenic selection of the IgVH repertoire, we used a data set from neonatal L2-TG mice [(35) see Materials and Methods] and repeated the microbial Blastp analysis. Although the IgVH CDR3 sequences from neonatal L2-TG mice demonstrated hits (i.e. exact matches, Figures 2A–C), this was nevertheless at a lower frequency than the IgVH CDR3 from DTG lupus mice (nine aa, 10% of that found in DTG; eight aa, 20%; seven aa, 50%), demonstrating an impact of mutation and diversification on identification of microbial mimics.

### Identification of matches between anti-DNA CDR3 and self proteins

**Figure 3:**
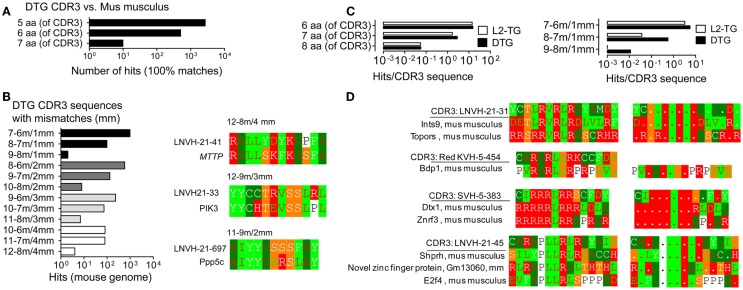
**Analysis of similarities of CDR3 with the *mus musculus* proteome**. Analysis of IgVH CDR3 sequences in Blastp with the mouse proteome. **(A)** Exact matches, DTG IgVH CDR3 sequences vs. contiguous sequences in the Mus musculus proteome. **(B)** DTG CDR3 vs. the *mus musculus* proteome: Left panel: Hits, i.e. matches with mismatches, denoted as in Figure 1. Right panels: Examples of matches with Microsomal triglyceride transfer protein large subunit isoform 2 precursor, MTTP (12–8 m/4 mm), Phosphoinositide-3-kinase, PIK3 (12–9/3 mm), Serine/threonine-protein phosphatase 5, Ppp5c (11–9 m/2 mm). **(C)** Hits (IgVH CDR3 vs. *mus musculus* proteome) normalized to number of IgVH CDR3 sequences from DTG or L2-TG mice. Left: 100% matches; right: matches including mismatches. **(D)** Anti-dsDNA IgVH CDR3 sequences from DTG with end stage lupus: Examples of hits, sequence matches/mismatches between individual IgVH CDR3 sequences from DTG lupus mouse and the mouse proteome. See also Figure 1 for details on color codes.

In the next analysis, we compared the IgVH CDR3 sequences from DTG lupus mice with the mouse proteome, see Materials and Methods. We found IgVH CDR3 with five, six, seven matches as well as examples of hits with mismatches (Figures 3A–C). As above, the mutated DTG sequences had higher frequency of matches. The frequencies of matches were 3–30× lower than those found toward microbial sequences (ratio of hits in mouse genome/hits in microbial genome. DTG: 28% of hits in the category seven aa/0 mm, 3% in eight aa/0 mm; corresponding results in L2-TG: 12%; 38%) as shown in Figures 3C and 1E.

The IgVH CDR3 sequences in the DTG data set are enriched for sequences that have anti-dsDNA specificity with a preponderance of positively charged arginines (R) that mediate binding to DNA. Perhaps not surprisingly, many of the hits in the mouse genome were DNA/RNA-associated proteins with matching positively charged residues (Figure 3D). To generalize and investigate which IgVH CDR3 aa were more likely to be associated with hits toward the mouse proteome, we analyzed the BALB/c data set. This includes >2000 sequences that have been downloaded from NCBI. These sequences include specificities toward diverse antigens and are more representative for global B cell responses and unbiased IgVH CDR3. With this data set we found that the IgVH CDR3 residues K, D, E and G, Y, L and multiples thereof (e.g. more than 1 K per IgVH CDR3 sequence) were positively associated with hits (Table S1 in Supplementary Material; see Materials and Methods for algorithm). IgVH CDR3 R was however only increased in hits if it was found more than 4× (i.e., CAR. R. R. R), when it was three times as likely to be found in the matched sequences. These results suggest that arginines *per se* (as overrepresented in the DTG data set) were not directly associated with enriched hits in the mouse genome.

### Similarities of IgVH CDR3 sequences with histone sequences

**Figure 4:**
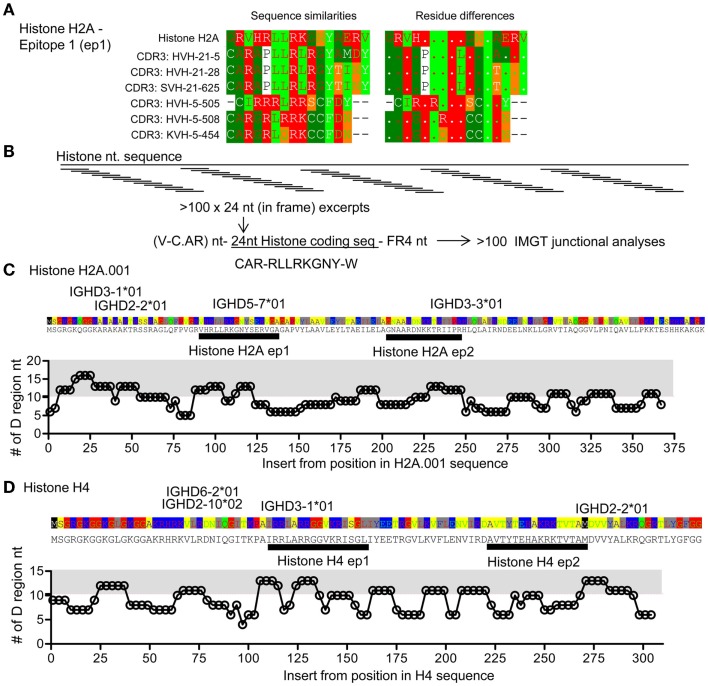
**Comparison of CDR3 and histone sequences; analysis of histone H2A sequence segments as part of V–D–J junctions**. **(A)** Examples of sequence comparisons of IgVH CDR3 sequences from DTG mice with end stage lupus and histone H2A, this epitope is denoted epitope 1 H2A-ep1. See also Figure S1 in Supplementary Material. **(B)** Artificial hybrid sequence files were made by inserting histone sequences – replacing the IgVH CDR3 to form model sequences. IgVH CDR3 regions are usually less than eight amino acids long, corresponding to 24 coding nucleotides. In frame H2A sequences were electronically generated i.e. HisH2A nt 1–24, nt 4–27, … , nt 367–391 and electronically engrafted onto a random IgVH-segment. A random FR4 was grafted onto this artificial IgVH – “Histone H2A-CDR3.” Resulting model sequence files were up-loaded for IgVH junctional analysis at IMGT. **(C)** The lengths of suggested IMGT D-segments are plotted. The average D segment usage in BALB/c mice is 10.2 nt. Gray area: >11 nt. HisH2A sequence is provided above the plot, possible D gene segments are positioned according to highest levels of matching. Two epitopes are marked, histone H2A-ep 1 [see also **(A)**] and histone H2A-ep2. See also Figure S1 in Supplementary Material. **(D)** Analysis of histone H4 corresponding to that performed in **(C)**. Two epitopes are marked histone H4-ep1 and histone H4-ep2, see also Figure 5 and Figure S1 in Supplementary Material.

The above analyses suggest that Th responses toward conventional autoantigen and Id may in fact be related in lupus. It has been suggested that histone-derived peptides constitute antigens for pathogenic Th cells in lupus (13). We therefore restricted the analysis to investigate IgVH CDR3 Id as potential mimics of histone sequences. Utilizing the sequences from the DTG mice with Id-driven lupus, we found IgVH CDR3 sequences with similarities to a histone H2A as well as H4, sequences previously identified as a Th cell antigens in lupus (13), Figure 4A. Similar matches were found for human IgVH CDR3 sequences when compared with human histone H2A and H4 (Figure S1 in Supplementary Material).

The above analyses searched for similarities between IgVH CDR3 sequences and genomic sequences, including histones. Reversing the analysis, we investigated if stretches of histone sequences could be coded by sequences similar to VDJ sequences, i.e., tested how well histone sequence excerpts could be accommodated within constraints of the VDJ junction. Focusing on histone H2A and histone H4, we pasted histone nt sequences onto a model IgVH, creating Vregion – histone segment-FR4 sequences. Resulting sequence files were uploaded into IMGT and subjected to VDJ junction analysis (Figure 4B; see Materials and Methods). With the histone H2A, we found that histone inserts could be coded by D genes at three sites, Figure 4B. Using such excerpts, the D region segment lengths were above average (10.3 nt, from analysis of >2000 BALB/c sequences, data not shown), Figure 4C. One of these epitopes corresponds to matches found with those described above (Figure 4A) called histone H2A epitope 1. Similarly, we identified four stretches of histone H4 that could be coded by other distinct D regions. One of these corresponded to the match seen in Figure 4A, histone H4, epitope 1.

### Anti-histone Th cell responses in DTG mice

**Figure 5:**
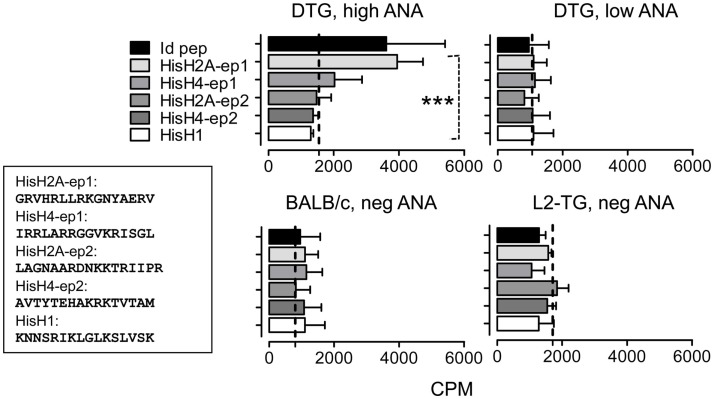
**Analysis of histone-specific Th cell responses in DTG mice and controls**. Based on the predictions in Figure 4 and Figure S1 in Supplementary Material, histone peptide stretches with similarities with mouse IgVH CDR3 sequences were tested in DTG lupus mice. Lymph node Th cells were from controls (bottom) or high and low serum ANA DTG mice were tested for responses toward Id-peptide, or histone peptides from HisH1, HisH2A, or HisH4 (see Materials and Methods and Figure 4 where His H2A and H4 peptides are shown) presented by irradiated BALB/c splenocytes. Dotted line: Control without peptide, *n* = 6. Upper left histograms (DTG, high ANA): One-way Anova, *p* < 0.0008, with Tukey’s Multiple Comparison test, *p* < 0.05 for His2A-ep1 vs. other histone peptides and control).

**Figure 6:**
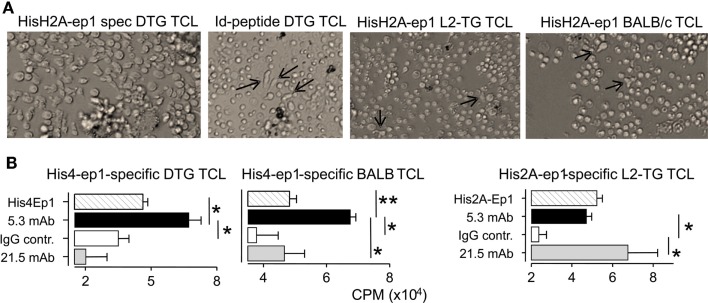
**Histone-specific T cell lines cross react to mAbs with CDR3 mimotopes**. Th cell lines were cultured with irradiated APC and peptides in 10-day cycles, see Materials and Methods. **(A)** Micrographs Th cell lines (TCL): Left second cycle Th cells from DTG mice responding to HisH2A-ep1 peptide, Th cell blasts and proliferation clusters are seen. Middle left: third cycle Th cells from DTG mice responding to Id-peptide, only few Th cells are seen (arrows) admixed with APC. Middle, right: third cycle His2A-ep1 TCL Th cell line from L2-TG mice, sparse Th cells (arrows) are indicated. Right: third cycle His2A-ep1 TCL from BALB/c. **(B)** Analysis of peptide specific TCL, cross reaction to mAbs: TCL lines from DTG, BALB/c and L2-TG were tested for responses toward mAbs with IgVH CDR3 sequence similarity with histone peptides. 5.3 mAb: IgVH CDR3 similar to His4Ep1. 21.5: IgVH CDR3 similar to His2A-ep1. Polyclonal mouse IgG was control. Shown is proliferative response toward appropriate peptide or mAbs (*n* = 4, Student’s *t*-test).

Following up the analyses above, we investigated if the lupus in DTG mice was associated with loss of tolerance toward histone motifs. In DTG mice with high ANA, but not in ANA-low DTG, we found significant responses toward both the H2A.001 and increased, but not significant response toward histone H4 epitopes (Figure 5). We proceeded to make Th cell lines towards each of these peptides. These were readily generated from DTG mice, but required more than five to six re-stimulations when Th cells were derived from single L2-TG or BALB/c mice (Figure 6A). The histone His4 ep1 (IRRLARRGGVKRISGL) has similarities with the IgVH CDR3 from the anti-DNA hybridoma 5.3 (CIRRRLRRSCFDYWG) but is dissimilar to IgVH CDR3 from the anti-DNA 21.5 (CARAPLLRLRGYAMDY). Strikingly, when purified mAb was used from the former, but not the latter, Th cell responses were elicited in Th cell lines specific for the H4 peptide (Figure 6B, and data not shown). Reciprocally, Th cells specific for H2A peptide (GRVHRLLRKGNYAERV) responded to the 21.5 mAb with CDR3 (CARAPLLRLRGYAMDY) and less to the 5.3 mAb, Figure 6B, and data not shown. The Th cell lines did not respond to Id-peptide from the transgene, data not shown. The anti-dsDNA mAbs were negative for Id^+^λ2^315^ L chains both in ELISA (data not shown) and by MALDI TOF mass spectrometry analysis (33).

## Discussion

We found that IgVH CDR3 from the mutated repertoire of lupus mice had a surprisingly high rate of matches toward the microbial proteomes, eukaryotic pathogens (fungi and protozoa), as well as self-proteins. Results suggested that these antigenic determinants may potentially stimulate common Th cell populations – and that these disparate peptide sources could constitute a network of peptide mimics. The frequency of matches was increased in the data set derived from the DTG mice with lupus, compatible with mutation and diversification of the autoimmune repertoire. Moreover, even with a limited set of IgVH CDR3 regions from mice with lupus (176 sequences), it was possible to identify exact matches from microbial proteomes of up to nine contiguous aa. We also found examples in categories such as 11 aa including 10 matched aa and 1 mismatch; or longer stretches of for example 15mers with 11 matches. Lupus mice with high levels of anti-dsDNA autoantibodies also had detectable Th cell responses toward histone determinants. Histone-reactive Th cell lines from such mice also responded with proliferation to stimulation when provided with anti-dsDNA mAbs with IgVH CDR3s that were similar to the histone epitopes, but did not respond to anti-dsDNA mAbs that had dissimilar CDR3s. Molecular mimics have been suggested in EAE (mice) and MS (humans), for example peptide mimics from pathogens were found to stimulate myelin basic protein (MBP)-specific Th cells (1, 2). However, these peptide epitope mimics had seven or five matched aa respectively across 15mer stretches (1, 2). The current findings of up to 11 matched aa in 15mers, and identity across 9mers suggest that idiotypes can provide a diverse pool of peptide mimics that may impact Th cell immunity.

The current results do not include analyses and prediction of peptide binding to MHC class II. TCR recognize peptides in the context of MHC, peptide mimicry could only occur if peptide sets could be presented to Th cells. In this regard, recent mathematical modeling and analysis of unbiased IgVH sequences from humans have revealed that V region peptides are especially well suited at binding MHC class II (37). The analysis demonstrated that the CDR regions had a unique propensity for MHC class II binding, and represent a frequent source of potential TCR exposed motifs (Th cell epitopes), (37).

It has previously been suggested that V region idiotypes may regulate immune responses, regulate clonal size, and also provide tonic signals for Th cells (15, 16, 18–22, 24, 25, 27–33, 37, 38). In the current setting, IgVH sequences were derived from lupus mice that developed a skewed oligoclonal repertoire enriched for cationic aa and especially arginines (33), which are aa that can mediate binding to dsDNA. These sequences were very similar to those found in other mouse models of lupus (33). We have previously demonstrated that Id-specific Th cells can induce anti-Histone autoantibodies (28) as well as anti-dsDNA antibodies, vasculitis, and nephritis (30, 31) and that both Th cells and B cells were necessary for development of the observed pathogenesis (30).

Lupus is associated with oligoclonal expansions of anti-dsDNA B cells (9, 11, 33, 39). B cells that are stimulated through the BCR in the absence of T cell help develop into anergic cells and undergo apoptosis, as reviewed in Ref. (40). Antigen specific Th cells can negate anergy and support conventional immune responses including germinal center reactions, development of plasma cells and autoantibody secretion (39, 41–43). Hence, anti-dsDNA B cell responses are dependent on help from Th cells, and both Th2 cells and follicular helper T cells have been directly linked to SLE (9, 11, 39).

In this setting, B cells will present a skewed repertoire of IgVH CDR3 peptides on MHC class II molecules to Th cells. This as B cells process and present endogenous BCR V regions on MHC class II to Th cells (18–20, 22, 25, 27–31, 33, 38). Idiotypes can also be presented by other antigen presenting cells, such as dendritic cells after receptor mediated endocytosis, and antigen processing (15, 16, 20–22, 24, 25, 28, 32, 38). In the case of lupus, uptake of immune complexes of dsDNA/anti-dsDNA could recruit Th responses directed against V region peptides. Recruitment would be facilitated by efficient antigen loading of immune complexes via Fc receptors, establishment of a pro-inflammatory microenvironment (serving as TLR ligands and inducing type I cytokines and chemokines) and contribute to a reduced regulatory activity, as has also been associated with Th cell responses directed toward antibody-based biologics (44).

The current results suggest that in settings with 1) skewed expression and presentation of endogenous V regions by B cells, 2) presentation of V regions from immune complexes by non-B cell APC, and 3) ongoing inflammation may lead to epitope spreading. Hence, it is possible that cationic CDR3 dsDNA-binding sequences may also provide mimics for Th cell responses directed against cationic peptides in dsDNA-associated proteins.

What is the potential for mimicry and TCR cross reactions? In general terms, TCRαβ diversity has been estimated to potentially exceed 10^20^ as a result of nucleotide insertions and somatic recombination of gene segments (45). However, estimates suggest that there are <10^8^ different antigen receptors in the naïve T cell pool (46). In any case, these numbers are dwarfed by the potential number of antigenic peptide-MHC molecules related to pathogens. Nevertheless, the relatively small number of TCRs provides an effective immune recognition to most pathogens. To bridge the gap of many orders of magnitude, it was suggested that TCR recognition may be degenerate (3, 4, 47). As a consequence, TCRs may be widely cross reactive. In fact, TCR cross reactivity has been implicated in both pathogenic and protective immunity in a number of diseases or infections (7, 48–52). TCR cross reactivity has recently been studied in context of class I (4) and class II (53). With regards to pMHC class I binding, the TCRαβ was estimated to potentially bind 10^6^ peptides (4). This is more than three orders of magnitude higher than that found for MHC class II by pMHC display libraries in yeast (53). TCR specificity resides in the arrangement of the variable CDR3 loop that may interact with TCR contact residues of the peptides (53). Cross reactive peptides could harbor interchangeable aa similars (53), as has also described earlier (5, 7). In terms of the current results, it is likely that the cationic CDR3 peptides enriched for DNA-binding could provide a potential mimic for cationic DNA-associated peptide sequences.

As anti-dsDNA antibodies share canonical properties across mouse models as well as between species, Th cell responses toward such idiotypes may be accompanied with the potential for responses toward seemingly not related DNA-associated proteins. For example, histone H4 (aa 71–94) has been described to be a Th cell epitope in lupus mice and in SLE patients. This peptide contains the following DNA-associated motif: HAKRKTVTAMD (13). The sequence is comparable to the IgVH CDR3 from that of several previously described anti-DNA IgVH CDR3 derived peptides (33). If Th cells respond to cationic Id peptides of such B cells, it could be hypothesized that established Th cell responses would potentially cross react to peptide mimics from other DNA-binding proteins, including histones as well as bacterial sequences with the same flavor (14).

In addition to this mimicry on the level of peptide presentation, B cell immune responses toward pathogens may initiate anti-DNA responses as demonstrated for pneumonia vaccines, and polysaccharide-binding antibodies (54, 55). In such situations expansion of anti-DNA B cells presenting a skewed CDR3 pMHC may result in Th responses that could cross react to a range of potential mimics. If so, the immune responses to these seemingly disparate peptides are related in Lupus.

## Conflict of Interest Statement

The authors declare that the research was conducted in the absence of any commercial or financial relationships that could be construed as a potential conflict of interest.

## Supplementary Material

The Supplementary Material for this article can be found online at http://journal.frontiersin.org/article/10.3389/fimmu.2015.00382

## Footnotes

^1^http://www.ncbi.nlm.nih.gov/nuccore/

^2^http://www.imgt.org/

^3^http://blast.ncbi.nlm.nih.gov/Blast.cgi

^4^http://athena.bioc.uvic.ca/virology-ca-tools/blast/

^5^http://www.nrbsc.org/gfx/genedoc
