# Supplementary material for: Systemic Lupus Erythematosus: Molecular Mimicry between Anti-dsDNA CDR3 Idiotype, Microbial and Self Peptides—As Antigens for Th Cells
Source: Front Immunol. 2015 Jul 28;6:382. doi: 10.3389/fimmu.2015.00382 (PMC4517057; doi:10.3389/fimmu.2015.00382)
Supplement: Supplementary file 1 [file Data_Sheet_1.PDF]

Supplementary Information

Supplementary Legends

S1. Supplementary Table 1. Analysis of amino acid usage in CDR3 sequences that have over 5 amino acid matches with the mus musculus proteome.

The table shows the likelihood of finding a particular aa in CDR3 sequences with hits (matches vs. the *mus musculus* proteome) as compared to the frequency of that aa in the >2000 BALB/c input CDR3 sequences, i.e. ratio of particular aa in hits vs. frequency of that particular aa in all input CDR3. Analysis also performed with 2, 3, 4 of a particular aa in hits vs. the same aa in all input CDR3. Overrepresented amino acids (>1.2) are indicated in underlined and bold.

# of aa acids/ CDR3	R	G	K	Y	E	D	F	W	V	H	P	L	I	A	T	Q	M	S	C	N
>1/CDR3	1.00	**1.29**	**1.44**	**1.22**	**1.44**	1.15	**1.26**	0.87	0.72	0.75	0.86	**1.56**	0.57	0.81	0.56	0.79	0.74	0.91	1.12	0.26
>2/CDR3	0.58	**2.22**	0.89	1.09	**2.42**	0.56	**1.53**	**2.88**	0.55	0.66	0	**2.40**	0	0.48	0.31	**1.67**	0	0.77	0	0.02
>3/CDR3	0.77	**4.75**	0	0.85	0.63	0.62	0	**3.75**	0	0	0	1.08	0	0.28	0.02	0	0	1.25	0	0
>4/CDR3	**2.98**	**4.43**	0	0.30	0	0	0	0	0	0	0	0	0	0.14	0.17	0	0	0	0	0

S2. Supplementary Figure S1. Comparison of histone with CDR3 sequences

A. Illustration of His4-ep1 as described by Datta (Datta, 2003) vs mouse IgG CDR3 sequences.

B. Analysis of human histone H2A inserted into human, i.e. VH- artificial “histone CDR3” – FR4 and uploaded to IMGT, junction analysis (vs. human VDJ). Analysis corresponds to the mouse sequences shown in Fig 4C.

C. Examples of sequence comparisons histone sequences vs. mouse, human and macaque VH CDR3.

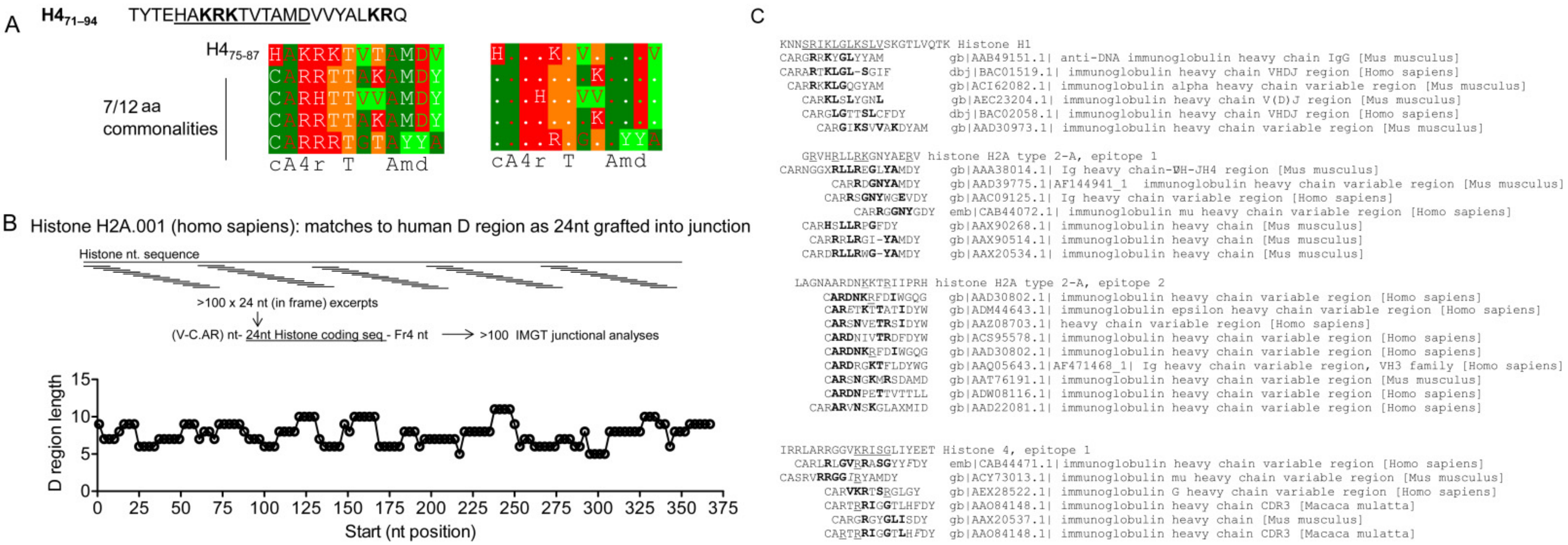

S3. Supplementary genome list

Genome List BlastP:

The microbial genome list was as follows:
http://blast.ncbi.nlm.nih.gov/Blast.cgi?PROGRAM=blastp&PAGE_TYPE=BlastSearch&BLAST_SPEC=MicrobialGenomes&LINK_LOC=blasttab&LAST_PAGE=blastp

Microbial (2117 databases, May 11, 2013). Microbial/222891 Microbial/784 Microbial/347255 Microbial/293614 Microbial/1105110 Microbial/33990 Microbial/788 Microbial/272944 Microbial/444612 Microbial/315456 Microbial/1032845 Microbial/652620 Microbial/35791 Microbial/1105114 Microbial/1105108 Microbial/562019 Microbial/481009 Microbial/782 Microbial/1105113 Microbial/783 Microbial/272951 Microbial/35794 Microbial/785 Microbial/263437 Microbial/307502 Microbial/163164 Microbial/77038 Microbial/384035 Microbial/77037 Microbial/1141110 Microbial/292805 Microbial/66084 Microbial/360910 Microbial/518 Microbial/519 Microbial/520 Microbial/94624 Microbial/152480 Microbial/95486 Microbial/1009846 Microbial/350701 Microbial/999541 Microbial/626418 Microbial/396598 Microbial/13373 Microbial/87883 Microbial/342113 Microbial/1229205 Microbial/391038 Microbial/398527 Microbial/28450 Microbial/882378 Microbial/269483 Microbial/640510 Microbial/640511 Microbial/640512 Microbial/516466 Microbial/416344 Microbial/987057 Microbial/1097668 Microbial/57975 Microbial/441166 Microbial/269482 Microbial/266265 Microbial/1070319 Microbial/266264 Microbial/106590 Microbial/977880 Microbial/887898 Microbial/391597 Microbial/576610 Microbial/264198 Microbial/329 Microbial/305 Microbial/658080 Microbial/658664 Microbial/243365 Microbial/546274 Microbial/888741 Microbial/887327 Microbial/629741 Microbial/557598 Microbial/888742 Microbial/546262 Microbial/546263 Microbial/484 Microbial/485 Microbial/486 Microbial/997348 Microbial/487 Microbial/488 Microbial/546267 Microbial/1032488 Microbial/547045 Microbial/665946 Microbial/641149 Microbial/546268 Microbial/1030841 Microbial/28091 Microbial/279714 Microbial/748280 Microbial/641147 Microbial/742159 Microbial/85698 Microbial/643561 Microbial/397945 Microbial/573060 Microbial/535289 Microbial/232721 Microbial/358220 Microbial/1036672 Microbial/179636

Microbial/76114 Microbial/62928 Microbial/748247 Microbial/469610 Microbial/522306 Microbial/33056 Microbial/1208919 Microbial/1208921 Microbial/1208920 Microbial/189385
Microbial/871271 Microbial/1005048 Microbial/285 Microbial/159087 Microbial/640081 Microbial/398578 Microbial/742013 Microbial/395494 Microbial/757424 Microbial/204773
Microbial/887062 Microbial/375286 Microbial/233181 Microbial/395495 Microbial/420662 Microbial/265072 Microbial/383631 Microbial/583345 Microbial/666681 Microbial/1000565
Microbial/582744 Microbial/887061 Microbial/228410 Microbial/335283 Microbial/153948 Microbial/261292 Microbial/323848 Microbial/847 Microbial/937450 Microbial/762966
Microbial/365044 Microbial/296591 Microbial/1007105 Microbial/365046 Microbial/338969 Microbial/987059 Microbial/983917 Microbial/580332 Microbial/742821 Microbial/84590
Microbial/29575 Microbial/85643 Microbial/292415 Microbial/75379 Microbial/426114 Microbial/34073 Microbial/565443 Microbial/391735 Microbial/543913 Microbial/314607 Microbial/161493
Microbial/404589 Microbial/447217 Microbial/862908 Microbial/959 Microbial/1184267 Microbial/693988 Microbial/563192 Microbial/1144275 Microbial/644282 Microbial/439235
Microbial/880072 Microbial/177437 Microbial/651182 Microbial/577650 Microbial/1167006 Microbial/96561 Microbial/485915 Microbial/525897 Microbial/706587 Microbial/555779
Microbial/177439 Microbial/643562 Microbial/690850 Microbial/207559 Microbial/876 Microbial/596151 Microbial/1121451 Microbial/573370 Microbial/879567 Microbial/411464
Microbial/526222 Microbial/457398 Microbial/665942 Microbial/298701 Microbial/881 Microbial/589865 Microbial/281689 Microbial/404380 Microbial/316067 Microbial/398767
Microbial/269799 Microbial/443143 Microbial/443144 Microbial/35554 Microbial/351605 Microbial/502025 Microbial/760142 Microbial/29546 Microbial/483219 Microbial/1278073
Microbial/246197 Microbial/338963 Microbial/338966 Microbial/391625 Microbial/448385 Microbial/378806 Microbial/335543 Microbial/56780 Microbial/262489 Microbial/88274
Microbial/28197 Microbial/572480 Microbial/944547 Microbial/391592 Microbial/195 Microbial/360104 Microbial/360105 Microbial/196 Microbial/553220 Microbial/360107 Microbial/197
Microbial/306263 Microbial/553218 Microbial/553219 Microbial/28080 Microbial/382638 Microbial/613026 Microbial/1002804 Microbial/537970 Microbial/138563 Microbial/213
Microbial/936155 Microbial/1216962 Microbial/235279 Microbial/679897 Microbial/537972 Microbial/210 Microbial/104628 Microbial/556267 Microbial/598659 Microbial/749222
Microbial/387092 Microbial/709032 Microbial/563040 Microbial/326298 Microbial/439483 Microbial/760154 Microbial/525898 Microbial/387093 Microbial/273121 Microbial/1249480
Microbial/598467 Microbial/9 Microbial/1240471 Microbial/203907 Microbial/291272 Microbial/859654 Microbial/572265 Microbial/1048758 Microbial/138073 Microbial/515618
Microbial/290338 Microbial/637910 Microbial/469595 Microbial/500640 Microbial/28141 Microbial/693216 Microbial/204038 Microbial/561229 Microbial/634503 Microbial/636
Microbial/1028307 Microbial/640513 Microbial/500639 Microbial/550 Microbial/888063 Microbial/399742 Microbial/469613 Microbial/693444 Microbial/552 Microbial/634500 Microbial/79967
Microbial/215689 Microbial/465817 Microbial/502347 Microbial/630626 Microbial/562 Microbial/564 Microbial/457400 Microbial/469598 Microbial/571 Microbial/573 Microbial/469608
Microbial/749535 Microbial/640131 Microbial/1124991 Microbial/553 Microbial/592316 Microbial/517433 Microbial/712898 Microbial/218491 Microbial/554 Microbial/1166016 Microbial/561231
Microbial/553480 Microbial/243265 Microbial/584 Microbial/471881 Microbial/520999 Microbial/521000 Microbial/500637 Microbial/588 Microbial/34038 Microbial/741091 Microbial/1286170
Microbial/218493 Microbial/28901 Microbial/615 Microbial/618 Microbial/768492 Microbial/399741 Microbial/768490 Microbial/768493 Microbial/138074 Microbial/621 Microbial/622
Microbial/623 Microbial/624 Microbial/556266 Microbial/343509 Microbial/51229 Microbial/406818 Microbial/406817 Microbial/527002 Microbial/349968 Microbial/630 Microbial/349966
Microbial/349965 Microbial/527012 Microbial/349967 Microbial/632 Microbial/633 Microbial/527004 Microbial/527005 Microbial/1191524 Microbial/1199246 Microbial/33059 Microbial/743299
Microbial/920 Microbial/872330 Microbial/470 Microbial/471 Microbial/707232 Microbial/575586 Microbial/575587 Microbial/575588 Microbial/436717 Microbial/40216 Microbial/62977
Microbial/525244 Microbial/575564 Microbial/575565 Microbial/878320 Microbial/380703 Microbial/382245 Microbial/998088 Microbial/393595 Microbial/930169 Microbial/236097
Microbial/187272 Microbial/572477 Microbial/156578 Microbial/28108 Microbial/715451 Microbial/374463 Microbial/422289 Microbial/422288 Microbial/207949 Microbial/114186
Microbial/91844 Microbial/413404 Microbial/412965 Microbial/638300 Microbial/290398 Microbial/167879 Microbial/314285 Microbial/777 Microbial/385025 Microbial/246195 Microbial/394104
Microbial/553217 Microbial/550540 Microbial/1163389 Microbial/264 Microbial/28110 Microbial/573569 Microbial/263 Microbial/1085623 Microbial/1129794 Microbial/983545 Microbial/455436
Microbial/349521 Microbial/768066 Microbial/999141 Microbial/349124 Microbial/555778 Microbial/314276 Microbial/283942 Microbial/1036674 Microbial/523791 Microbial/450 Microbial/446
Microbial/765910 Microbial/225937 Microbial/443152 Microbial/2743 Microbial/490759 Microbial/270374 Microbial/717774 Microbial/491952 Microbial/314277 Microbial/400668
Microbial/697282 Microbial/243233 Microbial/271065 Microbial/857087 Microbial/1026882 Microbial/754476 Microbial/754477 Microbial/637616 Microbial/480 Microbial/58051
Microbial/207954 Microbial/314278 Microbial/472759 Microbial/1229 Microbial/105559 Microbial/511062 Microbial/342610 Microbial/228 Microbial/234831 Microbial/87626 Microbial/259536
Microbial/335284 Microbial/1002339 Microbial/349106 Microbial/357804 Microbial/314282 Microbial/314283 Microbial/506534 Microbial/59196 Microbial/203122 Microbial/1033802
Microbial/326297 Microbial/62322 Microbial/314608 Microbial/318161 Microbial/318167 Microbial/458817 Microbial/323850 Microbial/211586 Microbial/398579 Microbial/24 Microbial/425104
Microbial/94122 Microbial/327275 Microbial/60480 Microbial/60481 Microbial/351745 Microbial/637905 Microbial/392500 Microbial/1117647 Microbial/762983 Microbial/377629 Microbial/
